# Supplementary material for: The RNA binding protein FgRbp1 regulates specific pre-mRNA splicing via interacting with U2AF23 in Fusarium
Source: Nat Commun. 2021 May 11;12:2661. doi: 10.1038/s41467-021-22917-3 (PMC8113354; doi:10.1038/s41467-021-22917-3)
Supplement: Supplementary file 1 — Supplementary Information [file 41467_2021_22917_MOESM1_ESM.pdf]

The RNA binding protein FgRbp1 regulates specific pre-mRNA splicing via interacting with U2AF23 in *Fusarium*

Minhui Wang[1], Tianling Ma[1], Haixia Wang[1], Jianzhao Liu[2], Yun Chen[1]*, Won Bo Shim[3]*, and Zhonghua Ma[1]*

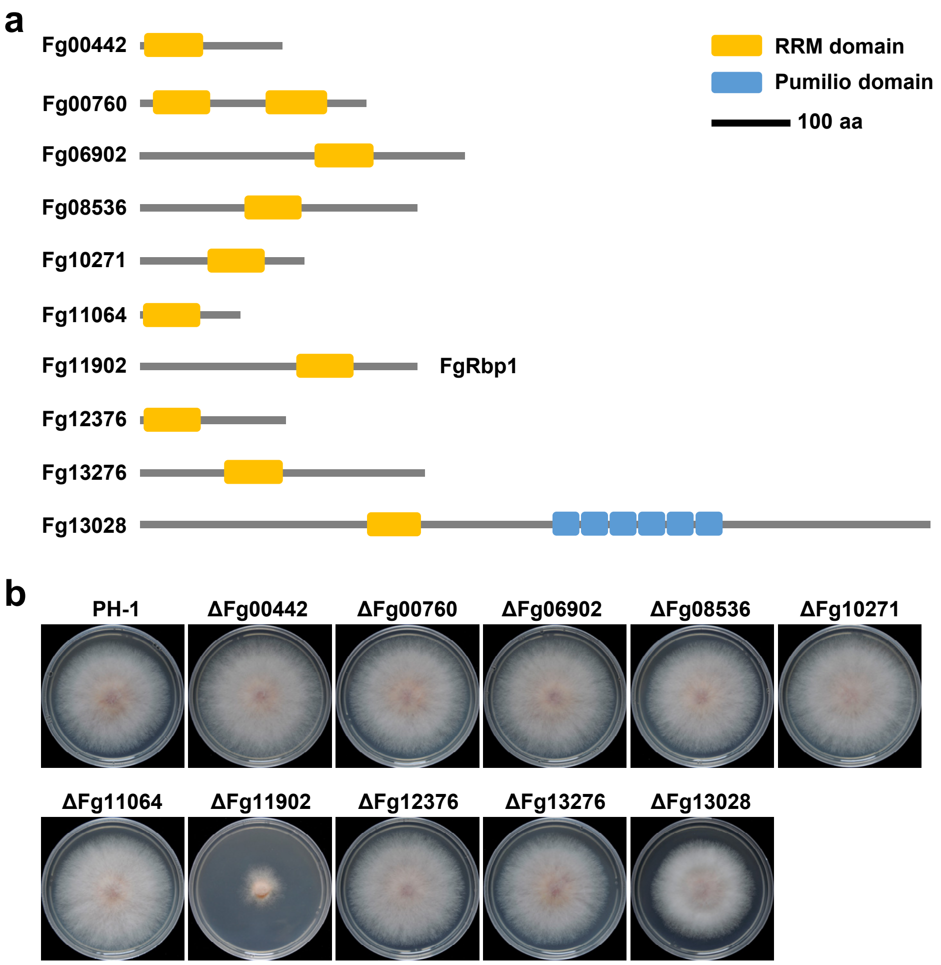

Supplementary Figure 1. Identification of uncharacterized RRM-containing proteins in *F. graminearum*. **a.** Schematic representation of domain organization of ten uncharacterized RRM-containing proteins. Individual domains are shown by different colors. **b**. Comparison of colony morphology of WT and ten RNA-binding protein mutants on PDA at 25°C for 3 d.

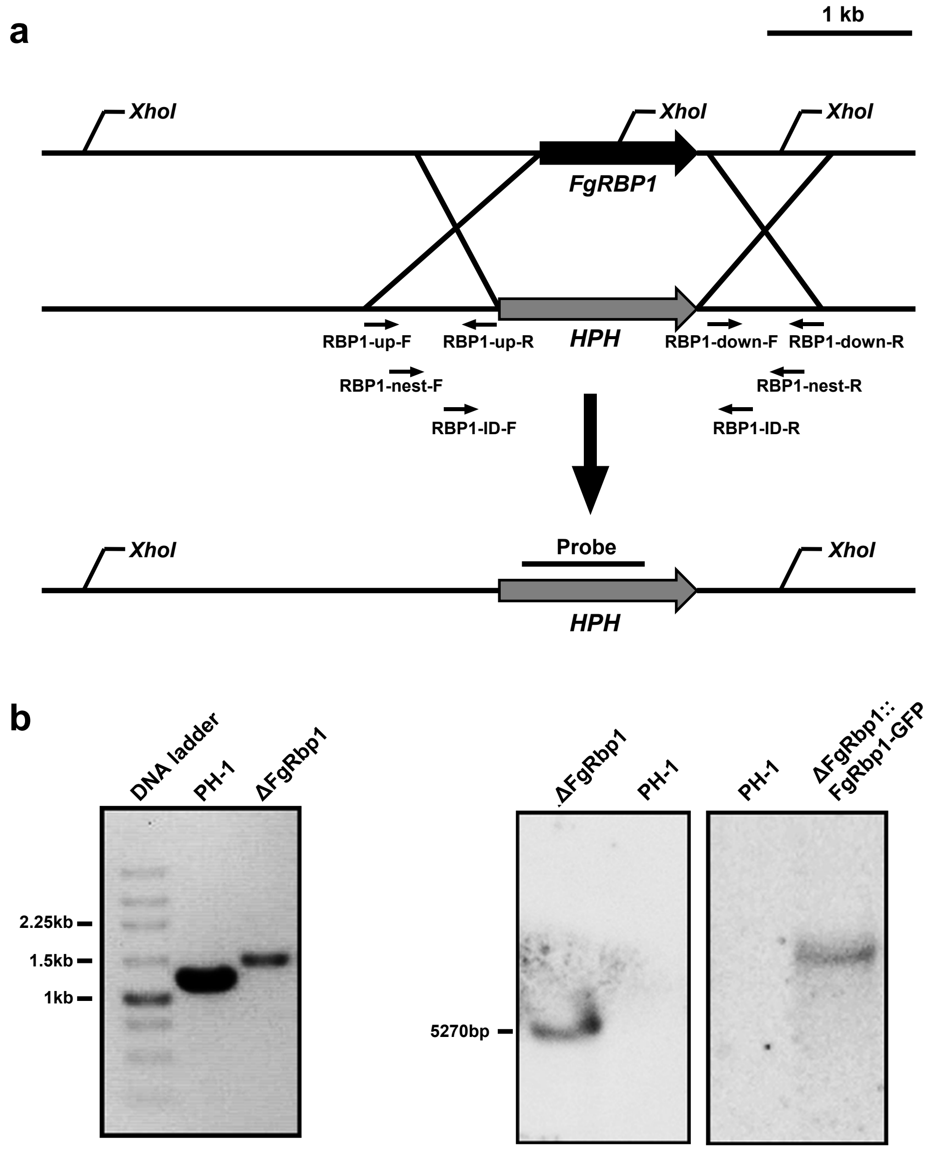

Supplementary Figure 2. Schematic representation of gene disruption strategy and Southern blotting of the deletion mutant ΔFgRbp1. **a.** Schematic representation of the *FgRBP1* disruption strategy. **b.** PCR assays for identification of *FgRBP1* gene deletion mutant. Using the primers shown in (**a**), a 1198-bp fragment was amplified in PH-1 and a 1491-bp fragment was amplified in the deletion mutant ΔFgRbp1. **c.** Southern blotting analyses of PH-1, ΔFgRbp1 and ΔFgRbp1::FgRbp1-GFP. The *HPH* fragment were used as the probe to identify ΔFgRbp1 and a 5270-bp fragment was hybridized in ΔFgRbp1. The NEO fragment was used as the probe to identify ΔFgRbp1::FgRbp1-GFP. A single fragment of unknown size was hybridized in the complemented strain.

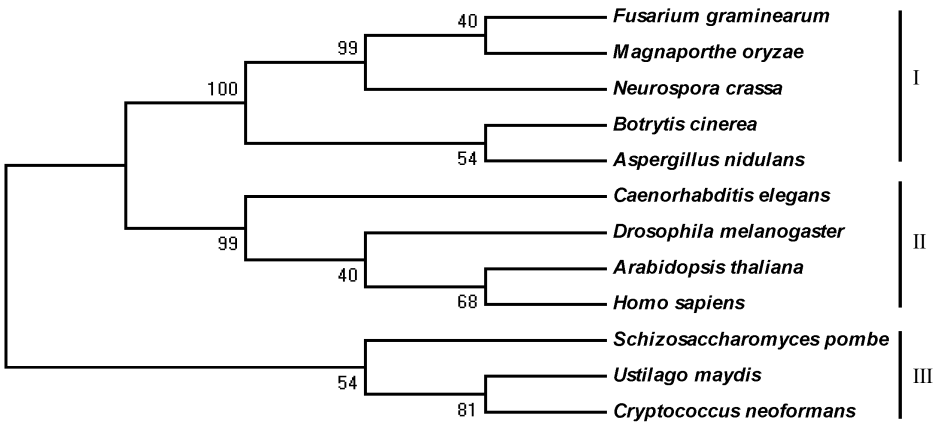

Supplementary Figure 3. FgRbp1 orthologs are conserved in fungi, metazoa and plants, but not in *Saccharomyces* species. The phylogenetic tree was constructed based on the amino acid sequences of FgRbp1 and its orthologs in other organisms with Mega 5.0 using the neighbor-joining method. The analyzed organisms include *Arabidopsis thaliana* (NP_187100.1), *Aspergillus nidulans* (XP_661557.1), *Botrytis cinerea* (XP_001558675.1), *Caenorhabditis elegans* (NP_498090.1), *Cryptococcus neoformans* (XP_012050974.1), *Drosophila melanogaster* (NP_649552.1), *Homo sapiens* (NP_077297.2), *Magnaporthe oryzae* (XP_003714189.1), *Neurospora crassa* (XP_964958.2), *Schizosaccharomyces pombe* (NP_594828.1), and *Ustilago maydis* (XP_011387189.1).

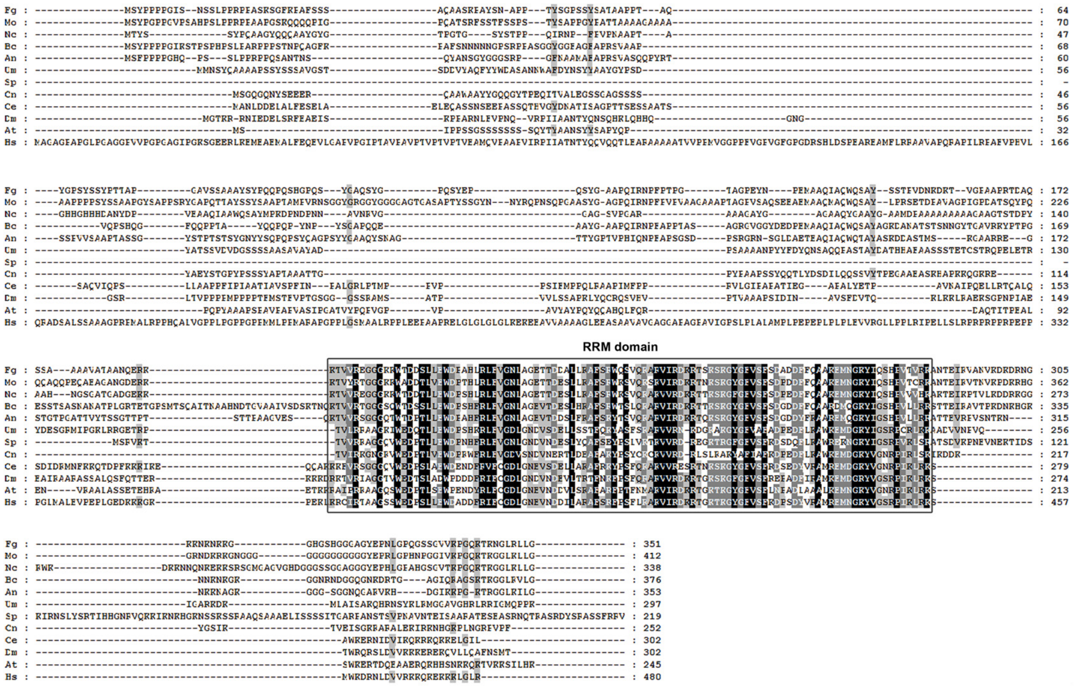

Supplementary Figure 4. Amino acid alignment of FgRbp1 orthologs reveals that the RRM domain of FgRbp1 is conserved among different species.

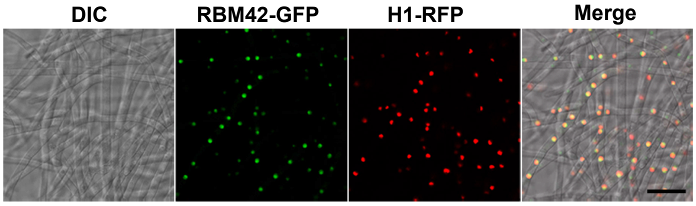

Supplementary Figure 5. Subcellular localization of RBM42-GFP in the strain ΔFgRbp1::RBM42-GFP::FgH1-RFP. Bar = 20 μm.

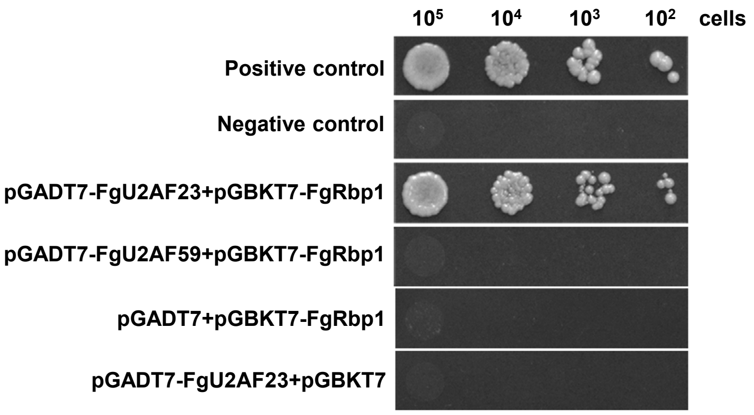

Supplementary Figure 6. The yeast two-hybrid (Y2H) assay indicated that FgRbp1 interacts with the small subunit of U2AF heterodimer (FgU2AF23), but not the large subunit (FgU2AF59). Ten-fold serial dilutions of yeast cells transferred with the bait and prey construct were assayed for growth on SD-Leu-Trp-His-Ade plates. A pair of plasmids, pGBKT7-53 and pGADT7-T was used as the positive control, while pGBKT7-Lam and pGADT7-T was used as the negative control.

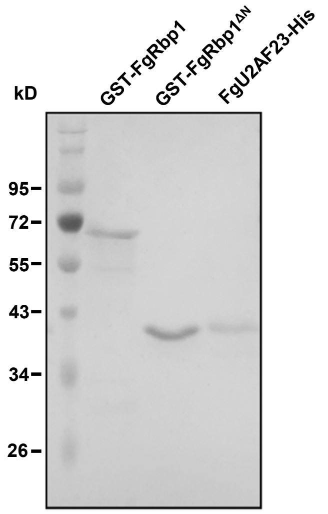

Supplementary Figure 7. GST-FgRbp1, GST-FgRbp1$^{\Delta N}$ and FgU2AF23-His were expressed in *E. coli*, purified using glutathione sepharose, resolved by SDS-PAGE on a 12.5% acrylamide gel, and stained with coomassie blue. The size of relevant molecular weight markers (MWM) are indicated on the left.

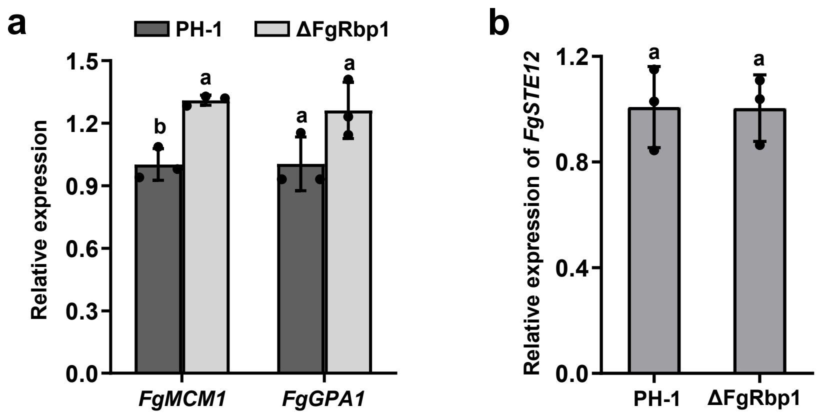

Supplementary Figure 8. Relative gene expression level of *FgMCM1*, *FgGPA1* **(a)** and *FgSTE12* **(b)** in PH-1 and ΔFgRbp1. Bars represent means ± SD (n=3 biologically independent replicates). Different letters indicate a significant difference ($P < 0.05$) according to the two-tailed unpaired Student's *t*-test.

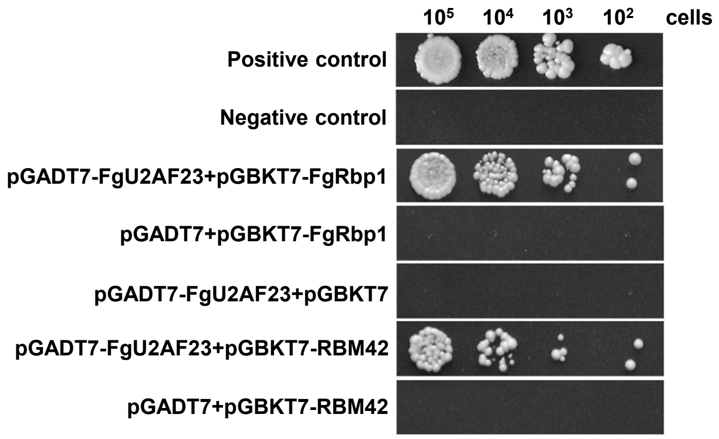

Supplementary Figure 9. Y2H assay revealed that human protein RBM42 interacts with *F. graminearum* protein FgU2AF23. Ten-fold serial dilutions of yeast cells transferred with the bait and prey construct were assayed for growth on SD-Leu-Trp-His-Ade plates. A pair of plasmids, pGBKT7-53 and pGADT7-T was used as the positive control, while pGBKT7-Lam and pGADT7-T was used as the negative control.

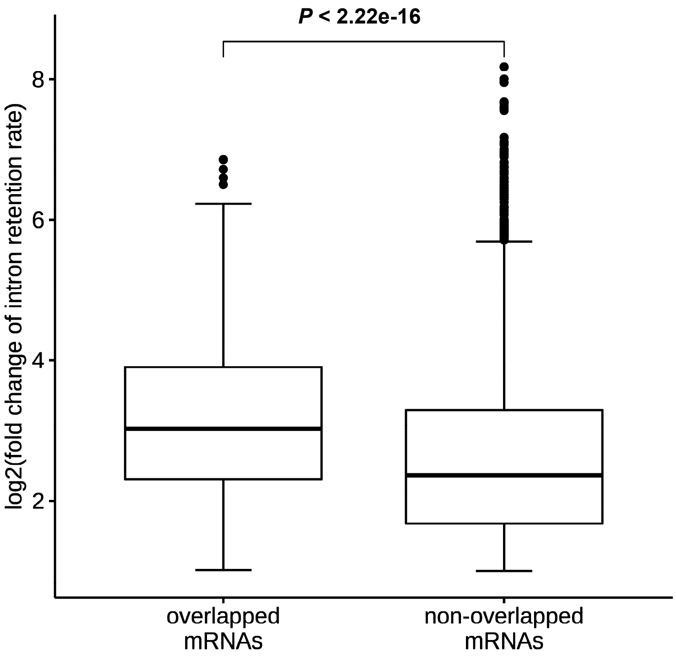

Supplementary Figure 10. Box plot of intron retention rates of FgRbp1-bound and unbound intron-retention genes. Data are represented as boxplots where the middle line is the median, the lower and upper hinges correspond to the first and third quartiles, the upper whisker extends from the hinge to the largest value no further than 1.5 × IQR from the hinge (where IQR is the interquartile range) and the lower whisker extends from the hinge to the smallest value at most 1.5 × IQR of the hinge, while data beyond the end of the whiskers are outlying points that are plotted individually. The y axis represents fold change of intron retention rate in ΔFgRbp1 over the WT strain. The x axis: 'overlapped mRNAs' represents intron-retention genes that overlap with RIP target genes (170 genes, as shown in Fig. 3d), 'non-overlapped mRNAs' represents the intron-retention genes excluded from RIP target genes (4679 genes, as shown in Fig. 3d). Statistical significance for the comparison was analyzed by two-tailed *t*-test ($P < 2.22e-16$).

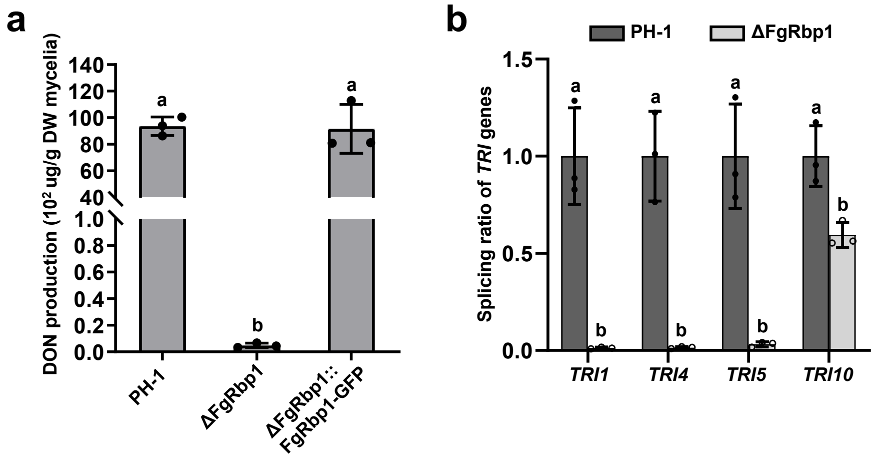

Supplementary Figure 11. FgRbp1 regulates the splicing of deoxynivalenol (DON) biosynthesis genes in *F. graminearum*. **a.** The FgRbp1 deletion mutant showed significantly reduced DON production in the mycotoxin-inducing medium (TBI). **b**. The splicing ratios of DON biosynthesis genes (*TRI* genes) in PH-1 and ΔFgRbp1. Bars represent means ± SD (n=3 biologically independent replicates). Different letters indicate a significant difference ($P < 0.05$) according to the two-tailed unpaired Student's *t*-test.

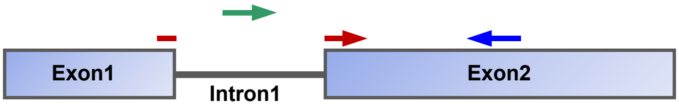

Supplementary Figure 12. Schematic representation of primers used for amplifying spliced and unspliced mRNAs for a specific intron. For amplifying the spliced RNA, the forward primer was designed to cross exon-exon junctions to ensure specific amplification of spliced RNA (as the red arrow depicts), and the reverse primer was designed on the exon adjacent to the intron (as the blue arrow depicts). For amplifying the unspliced RNA, the forward primer was designed within the intron (as the green arrow depicts), and the reverse primer was the same as that for amplifying the spliced RNA (as the blue arrow depicts).
