# Supplementary material for: The RNA binding protein FgRbp1 regulates specific pre-mRNA splicing via interacting with U2AF23 in Fusarium
Source: Nat Commun. 2021 May 11;12:2661. doi: 10.1038/s41467-021-22917-3 (PMC8113354; doi:10.1038/s41467-021-22917-3)
Supplement: Supplementary file 8 — Description of Additional Supplementary Files [file 41467_2021_22917_MOESM8_ESM.docx]

Description of additional supplementary information

Title: Supplementary Data 1

Description: RRM domain-containing proteins in Fusarium graminearum.

Title: Supplementary Data 2

Description: A list of putative FgRbp1-interacting proteins identified by yeast two-hybrid screens.

Title: Supplementary Data 3

Description: Intron retention genes in ∆FgRbp1

Title: Supplementary Data 4

Description: Putative target mRNAs of FgRbp1 identified by RIP- Seq.

Title: Supplementary Data 5

Description: A list of PCR primers used in this study and their relevant characteristics.
