# Supplementary material for: The RNA binding protein FgRbp1 regulates specific pre-mRNA splicing via interacting with U2AF23 in Fusarium
Source: Nat Commun. 2021 May 11;12:2661. doi: 10.1038/s41467-021-22917-3 (PMC8113354; doi:10.1038/s41467-021-22917-3)
Supplement: Supplementary file 9 — Reporting Summary [file 41467_2021_22917_MOESM9_ESM.pdf]

nature research

Corresponding author(s): Zhonghua Ma

Last updated by author(s): Mar 25, 2021

Reporting Summary

Nature Research wishes to improve the reproducibility of the work that we publish. This form provides structure for consistency and transparency in reporting. For further information on Nature Research policies, see Authors & Referees and the Editorial Policy Checklist.

Statistics

For all statistical analyses, confirm that the following items are present in the figure legend, table legend, main text, or Methods section.

n/a	Confirmed	
☐	☒	The exact sample size (*n*) for each experimental group/condition, given as a discrete number and unit of measurement
☐	☒	A statement on whether measurements were taken from distinct samples or whether the same sample was measured repeatedly
☐	☒	The statistical test(s) used AND whether they are one- or two-sided<br>*Only common tests should be described solely by name; describe more complex techniques in the Methods section.*
☒	☐	A description of all covariates tested
☒	☐	A description of any assumptions or corrections, such as tests of normality and adjustment for multiple comparisons
☐	☒	A full description of the statistical parameters including central tendency (e.g. means) or other basic estimates (e.g. regression coefficient) AND variation (e.g. standard deviation) or associated estimates of uncertainty (e.g. confidence intervals)
☐	☒	For null hypothesis testing, the test statistic (e.g. *F*, *t*, *r*) with confidence intervals, effect sizes, degrees of freedom and *P* value noted<br>*Give P values as exact values whenever suitable.*
☒	☐	For Bayesian analysis, information on the choice of priors and Markov chain Monte Carlo settings
☒	☐	For hierarchical and complex designs, identification of the appropriate level for tests and full reporting of outcomes
☒	☐	Estimates of effect sizes (e.g. Cohen's *d*, Pearson's *r*), indicating how they were calculated

Our web collection on statistics for biologists contains articles on many of the points above.

Software and code

Policy information about availability of computer code

Data collection	Confocal microscopic images were aquired with a Zeiss LSM780 confocal microscopy.<br>Real time PCR data were collected with Eppendorf RealPlex2 RealTime PCR System.
Data analysis	Clean reads were mapped to the F. graminearum genome by Hisat2 (version 2.1.0). Statistical analyses were performed using GraphPad PrismGraphs (version 8.0.1) , SAS (Statistical Analysis System, version 9.0), Image J (version 1.8.0), Microsoft excel 2007.

For manuscripts utilizing custom algorithms or software that are central to the research but not yet described in published literature, software must be made available to editors/reviewers. We strongly encourage code deposition in a community repository (e.g. GitHub). See the Nature Research guidelines for submitting code & software for further information.

Data

Policy information about availability of data

All manuscripts must include a data availability statement. This statement should provide the following information, where applicable:

- Accession codes, unique identifiers, or web links for publicly available datasets
- A list of figures that have associated raw data
- A description of any restrictions on data availability

Data supporting the major findings of this work are available within the paper and its Supplementary Information files. The RNA-seq data, RIP-seq data has been deposited in the NCBI SRA database with accession codes PRJNA556958 (https://www.ncbi.nlm.nih.gov/sra/?term=PRJNA556958) and PRJNA556748 (https://www.ncbi.nlm.nih.gov/sra/?term=PRJNA556748), respectively. All relevant data are available from the corresponding authors upon any reasonable request. Source data are provided with this paper.

Field-specific reporting

Please select the one below that is the best fit for your research. If you are not sure, read the appropriate sections before making your selection.

☒ Life sciences ☐ Behavioural & social sciences ☐ Ecological, evolutionary & environmental sciences

For a reference copy of the document with all sections, see nature.com/documents/nr-reporting-summary-flat.pdf

Life sciences study design

All studies must disclose on these points even when the disclosure is negative.

Sample size	Sample sizes are noted in the Figure legends or methods section. No statistical methods were used to predetermine sample size. Comparable sample sizes were chosen that allow data reproducibility for each experimental conditions. For all data, positive and negative controls were included whenever possible. The sample sizes provided for each experiment are typical and determined based on previous published researches (Tang GF et al., PLoS Pathog, 2018; Chen Y et al., Nat commun, 2018; Liu N et al., New phytol, 2019).
Data exclusions	No data were excluded.
Replication	All attempts at replication were successful. The number of replicates is indicated in the corresponding figure legend and/or in the corresponding "Methods" section.
Randomization	Randomization was not appropriate for this study as none of the experiments described in this study involve group allocation.
Blinding	Blinding was not appropriate for this study as none of the experiments described in this study involve group allocation during data collection or analysis.

Reporting for specific materials, systems and methods

We require information from authors about some types of materials, experimental systems and methods used in many studies. Here, indicate whether each material, system or method listed is relevant to your study. If you are not sure if a list item applies to your research, read the appropriate section before selecting a response.

Materials & experimental systems

n/a	Involved in the study	
☐	☒	Antibodies
☒	☐	Eukaryotic cell lines
☒	☐	Palaeontology
☒	☐	Animals and other organisms
☒	☐	Human research participants
☒	☐	Clinical data

Methods

n/a	Involved in the study	
☒	☐	ChIP-seq
☒	☐	Flow cytometry
☒	☐	MRI-based neuroimaging

Antibodies

Antibodies used	All antibodies used in this study are commercially available, including anti-GFP antibody (ab32146, Abcam, Cambridge, UK), anti-RFP antibody (ab65856, Abcam, Cambridge, UK), anti-Flag M2 antibody (Sigma, F1804), anti-GAPDH antibody (EM1101, HuaAn Biotechnology, Hangzhou, China), anti-RPS3 antibody (ET1601-27, HuaAn Biotechnology, Hangzhou, China) , anti-RPS20 antibody (ET1610-83, HuaAn Biotechnology, Hangzhou, China), anti-H3 antibody (ab8895, Abcam, Cambridge, UK), anti-GST antibody (EM80701, HuaAn Biotechnology, Hangzhou, China) and anti-His antibody (ab18184, Abcam, Cambridge, UK). The anti-Flag M2 antibody (Sigma, F1804) was used for the RIP assay.
Validation	The anti-GFP, anti-RFP, anti-GAPDH, anti-flag antibodies were previously validated (Tang GF et al., PLoS Pathog, 2018; Chen Y et al., Nat commun, 2018; Liu N et al., New phytol, 2019;). The anti-H3, anti-GST and anti-His antibody were previously validated (Chen Y et al., Nat commun, 2018). The anti-Flag M2 antibody has been used for RNA immunoprecipitation previously in several studies (Mukherjee, N et al., Mol Cell, 2011; Chen XS et al., Plant Cell, 2018). The anti-RPS3 and anti-RPS20 were certified and validated by manufacturers (https://www.huabio.com/products_pro_1982.html, https://www.huabio.com/products_pro_2250.html).
